# Reaching Out to Rural Caregivers and Veterans with Dementia Utilizing Clinical Video-Telehealth

**DOI:** 10.3390/geriatrics3020029

**Published:** 2018-06-09

**Authors:** James S. Powers, Jennifer Buckner

**Affiliations:** 1 Vanderbilt University School of Medicine, 7159 Vanderbilt Medical Center East, Nashville, TN 37232, USA; 2 The Geriatric Research Education and Clinical Center, Nashville TN 37212, USA; 3 The Tennessee Valley Healthcare System (JB) Nashville TN 37212, USA; jennifer.buckner@va.gov

**Keywords:** telehealth, dementia, care giver support

## Abstract

Context: A clinical video telehealth (CVT) program was implemented to improve access and quality of dementia care to patients and their caregivers in rural areas. The program was offered as part of an established dementia clinic/geriatric primary care clinic in collaboration with five community-based outpatient clinics (CBOC’s) affiliated with the Tennessee Valley Healthcare System (TVHS) in middle Tennessee. Telehealth support was provided by a physician–social worker team visit. Methods: Telehealth training and equipment were provided to clinic personnel, functioning part-time with other collateral clinical duties. Patients and caregivers were referred by primary care providers and had an average of one to two CVT encounters originating at their local CBOC lasting 20 to 30 min. Clinical characteristics and outcomes of patients and caregivers receiving CVT support were collected by retrospective electronic medical record (EMR) review. Results: Over a 3-year period 45 CVT encounters were performed on patient–caregiver dyads, followed for a mean of 15 (1–36) months. Some 80% patients had dementia confirmed and 89% of these had serious medical comorbidities, took an average of eight medications, and resided at a distance of 103 (76–148) miles from the medical center. Dementia patients included 33% with late stage dementia, 25% received additional care from a mental health provider, 23% took antipsychotic medications, 19% transitioned to a higher level of care, and 19% expired an average of 10.2 months following consultation. Significant caregiver distress was present in 47% of family members. Consult recommendations included 64% community-based long-term care services and supports (LTSS), 36% medications, and 22% further diagnostic testing. Acceptance of the CVT encounter was 98%, with 8770 travel miles saved. Conclusions: CVT is well received and may be helpful in providing dementia care and supporting dementia caregivers to obtain LTSS for high-need older adults in rural areas.

## 1. Introduction

### 1.1. Background

Alzheimer’s disease affects 47 million individuals worldwide, including 5 million individuals and 15 million caregivers in the United States [1]. Dementia patients are increased utilizers of health services, with a 57% increase in healthcare costs in the last five years of life [2]. Progressive disability and higher mortality associated with dementia care require supportive and proactive healthcare models to address these issues. Proactive medical and psychosocial care management improves quality of life and other health outcomes and increases quality of care by adherence to guidelines and referrals to community long-term care services and supports (LTSS) [3]. Caregiver distress is common among dementia caregivers, [4] and there is a great need to engage and support dementia family caregivers [5]. New models of care delivery to enhance access and person-centered care for veterans with dementia and their family caregivers are desperately needed.

The Tennessee Valley Healthcare System (TVHS) consists of two campuses separated by 40 miles, Nashville and Murfreesboro, and is located in Veterans Integrated Service Area 9 in Southeast US. A market analysis revealed that the Tennessee Valley Nashville campus had a census of over 1000 military veteran patients greater than age 80, 35% of whom resided in rural areas.

In 2011, the Nashville campus developed a geriatric patient-centered medical home model for geriatric primary care—the Geriatric Patient-Aligned Care Team (*Geri*PACT). The *Geri*PACT Team consists of the *Geri*PACT provider (geriatrician or geriatric nurse practitioner with an outpatient panel size of approximately 800), a social worker, a clinical pharmacist, a registered nurse care manager, a licensed vocational nurse, and clerical staff. These individuals were experienced in working as a coordinated unit delivering patient-centered assessments and managing medically complex and vulnerable elderly individuals. *Geri*PACT is a special population PACT for complex geriatric and other high-risk vulnerable veterans providing integrated, interdisciplinary assessment and longitudinal management, and coordination of both VA sponsored and non-VA sponsored (Medicare and Medicaid) services for patients and caregivers [6]. The clinic also provides geriatric assessment, falls assessment and treatment, and dementia consults.

Telehealth is especially critical in rural and other remote areas that lack sufficient health care services, including specialty care. The Office for the Advancement of Telehealth [7] promotes the use of telehealth technologies for health care delivery, education, and health information services. Clinical video-telehealth (CVT) has been used to deliver consult and team-based care for specialty care services and to improve hospital transitions of care [8,9,10]. This technology has been found to improve caregiver knowledge and support, enhancing the quality of dementia care by fostering close collaboration relationships with staff from other referring clinics for reliable follow-up and completion of recommended actions [11].

We report our innovative application of real-time video technology coupled with care management and principles of team care, utilizing the electronic health record (EHR) to deliver dementia care to rural veterans in the TVHS service area.

### 1.2. Context and Development of the Model

**Figure 1:**
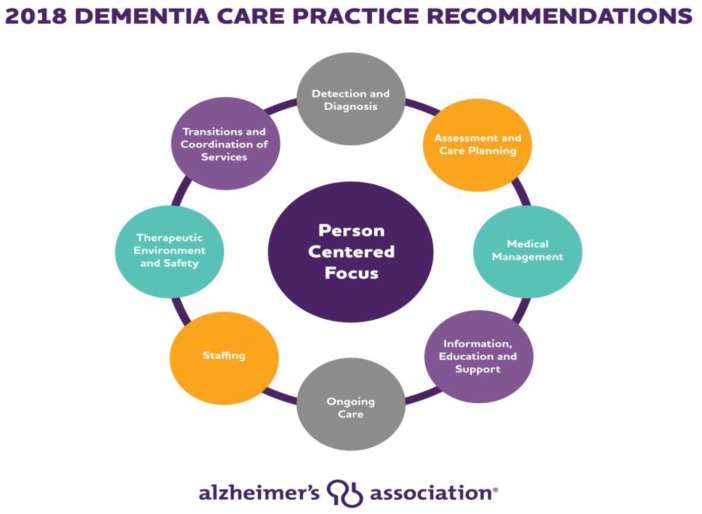
Dementia Care Practice Recommendations [1] (reproduced with permission).

Our program was developed to improve access and quality of person-centered dementia care to patients and their caregivers who live in rural areas by providing distance dementia care and caregiver support which included CTV. This project involved collaboration with five community-based outpatient centers (CBOC’s) affiliated with TVHS offered as part of the established Dementia Consult/Geriatric Primary Care Clinic. CBOC’s share the same electronic health record and Veterans Health Administration policies and procedures. Telehealth support was provided to patients and caregivers seen simultaneously by a Nashville-based physician and social worker team visit, to provide person-centered dementia care consistent with current standards of care practice recommendations (Figure 1).

As a component of *Geri*PACT, the dementia clinic provided 60 consultations per year and performed approximately 10 e-consults (provider-requested chart reviews with no patient contact) for patient–caregiver dyads seen at affiliated rural CBOC’s for dementia patients with transportation difficulties. There are 14 CBOC’s affiliated with TVHS, varying between 50 to 148 miles distance from the medical center. The dementia clinic provided medical diagnostic evaluation and management as well as engaging home and community-based long-term care services and supports (LTSS) that were person-centered and tailored to individual needs of the patient and caregiver. This included, when indicated, skilled services such as home health care, nursing, physical and occupational therapy, home safety evaluations, and adaptive durable medical equipment. For eligible patients, it also included non-skilled services such as homemaker home health aides, respite care, and adult day services through state Medicaid and VA programs. Additional community supports were also made available such as information and referral, assistance with transportation, and transitions to higher levels of care appropriate to patient status. These non-skilled community support services help to decrease social isolation and provide family and caregiver support and education to make informed choices about care.

## 2. Methods and Process Improvement Implementation of the Model

In implementing thepatient-centered medical home, we followed the Promoting Action on Research Implementation (PARIHS) Framework [12,13] which maintains that implementation processes commonly include five elements: Planning and modeling the program, engaging support from collaborators funders and administration, executing or implementing the new process, reflecting on progress both-successes and barriers, and process and outcome evaluation. 

### 2.1. Planning

Over an 18-month period potential CBOC’s, were contacted and visited by the *Geri*PACT team to offer CVT services. A relationship was formed with five sites, and the program developed template notes, service agreements, and addressed staff training and confidentiality. Equipment needs including cameras and monitors were obtained through VA Office of Rural Health funding.

### 2.2. Engaging Support

CBOC providers, RN care managers, and social work staff were encouraged to refer panel dementia patients and caregivers and appreciated assistance with LTSS counseling and referrals. Three-year partial rural health funding was necessary to partially support staffing essential to the patient centered medical home function. This enhanced administrative buy-in for the expanded part-time consult clinic.

### 2.3. Executing

All clinic personnel had collateral duties and the physician director covered messages, received referrals, and directed scheduling. Dementia patient–caregiver dyads were referred by CBOC providers as well as nursing staff and social workers. The CVT visits were scheduled and delivered by computer screen at the patient’s local community-based clinic. The duration of CVT encounters averaged 20–30 min and consisted of a single visit with an occasional second visit as requested by providers and families. Patient interview, EHR review, and caregiver assessment were performed during the encounter. Recommendations were given to the provider, including further diagnostic testing, medication reconciliation and consultation, and LTSS. The videoconferencing was secure, no recordings were made, and a summary of the consultation was documented in the electronic medical record (EMR) including the history, evaluation, recommendations, and referrals performed. Referrals for community-based services and supports were initiated by the consulting physician or social worker including caregiver education regarding available home and community-based resources, disease course and prognosis, advance care and long-term care planning, and coordination of services. The Veterans Health Administration provides many non-skilled services. Along with discussing in-home and community supports and services, veterans and caregivers were educated on the Shared Decision Making resource tool [14]. This electronic and print resource is a person-centered tool that is able to provide veterans and caregivers with information to assist them with making difficult choices, such as long-term care. It explains the full array of VA Geriatrics and Extended Care (GEC) services, eligibility, and processes, and provides a platform for decision-making regarding person-centered geriatric care. The consult team continued to serve as a resource for the primary care provider with availability of between-visit follow-up telephone and e-consultation. All providers had access to the same EHR, the VA computerized patient record system (CPRS) for consultation entries, requests, documentation, and follow-up e-consults and communications.

### 2.4. Reflecting and Evaluating

Clinical characteristics of patients and families receiving CVT support were collected from March 2015 to March 2018 including: age, location of residence, diagnosis, functional status, caregiver characteristics, consultation recommendations, and community services arranged. Dementia severity was assessed by the Functional Assessment Staging Test (FAST) scale (4 early, 5 mid-stage, 6–7 late-stage) which could be readily assessed from information obtained by the EHR and during the CVT visit [15]. Patients were referred for formal neuropsychiatric testing when clinically indicated at the discretion of the primary care provider. Patients and caregivers were asked about their satisfaction with the CVT visit, and caregiver distress was determined from self-report during the encounter.

The Tennessee Valley Healthcare System Institutional Review Board acknowledged this project as a quality improvement initiative, intended for practical, clinical, and administrative purposes. All patients agreed to clinical treatment which included telehealth modalities.

## 3. Results

Over a 3-year period 45 CVT encounters were performed on patient–caregiver dyads. (Table 1) CVT visits grew to comprise 25% of the *Geri*PACT dementia consults. Over this time e-consult requests from CBOC’s for chart review only declined. Retrospective EHR follow-up for these patients was a mean of 15 (1–36) months. When asked about their acceptance of the video consultation, patients and caregivers expressed great satisfaction during the CVT encounter experience (98%), as well as the convenience of being seen close to home. Mean distance from the consult clinic was 103 (76–148) miles, representing an actual total 8770 transportation miles saved over the time frame of the pilot. Families appreciated receiving care closer to home, and avoiding travel with dementia patients exhibiting behavioral problems. A total of 20% of visits revealed no dementia, however, these patients had significant mental health comorbidities including post- traumatic stress disorder, depression, and bipolar disease (78%). Non-dementia patients also tended to be younger, with a mean age of 71 (54–85) years. Dementia patients represented 80% of the consults and 89% had significant medical comorbidities, 44% had two or more activities of daily living (ADL) deficits, took an average of eight medications, and 44% had advanced care planning documents in place. Mean age was 77 (63–100) years, 50% had mid-stage and 33% had late-stage vascular or Alzheimer’s type dementia, 25% received other mental health services, and 23% took antipsychotic medications. Consult recommendations included: LTSS in 23 patients (64%), medications in 13 patients (36%), and further diagnostic testing in eight patients (22%). Clinic staff helped to arrange LTSS on behalf of CBOC patients and referring providers.

Some 19% of dementia patients transitioned to a higher level of care (assisted living, dementia care unit, or nursing home), and similarly 19% expired within a mean 10.2 months following consultation. Among caregivers, 70% were spouses, 30% were other family members. Some 44% of patients met the definition of high need older adults—those with dementia and two or more ADL deficits. Caregiver stress was present in all, and 47% of caregivers additionally reported high levels of distress including obvious anxiety, burnout, tearfulness, and worsening health observed directly during the encounter. 

## 4. Discussion and Implications

The CVT model may be a value-added means to manage frail, high-need elderly patients, contributing to cost avoidance and improved outcomes. Patients and caregivers as well as providers appreciated the convenience and timeliness of the consults, and requests for CVT consults grew to comprise 25% of all *Geri*PACT dementia consults. 

Damschroeder et al. [16] suggest that implementation science may be understood by utilizing a consolidated framework including: the intervention characteristics, both external and internal context, participant characteristics, and the implementation process. A consolidated framework for the implementation process for the CVT Dementia Clinic included the following: (1) The intervention characteristics involved planning over an 18-month time frame, was data driven, and utilized a person-centered dementia care conceptual model; (2) market data and patient characteristics provided both external and internal context; (3) participants engaged support by visiting clinics and communicating with local CBOC telehealth coordinators; (4) the implementation process involved developing templates, providing staff training and equipment, and refined logistics for scheduling and delivering for team-based visits.

CVT technology and interoperability of the same EHR facilitated delivery of services close to home, where patients and caregivers live. These patients resided an average of 100 miles away from the medical center. The majority had major medical comorbidities and over one third had advanced dementia. Nearly 20% died within one year of the consult and nearly half of caregivers reported serious caregiver distress. Clinic staff facilitated obtaining LTSS for over half of patient–caregiver dyads.

### 4.1. Replication and Future Plans

CVT was developed in a non-fee-for-service system which valued cost avoidance, demonstrating decreased utilization of resources with 8770 travel miles saved at an estimated $0.415/mile = $3240. Expansion of CVT consults may be helpful in extending our reach for other medical centers and could be utilized for other specialties such as mental health and palliative care services. New technology has now made possible Home-CVT, a secure Skype-like connection between patients, caregivers, and the specialty clinic. This application permits patients to be seen remotely in the home setting, avoiding the need for transportation to the clinic.

### 4.2. Limitations

Development of CVT for dementia care occasionally strained local CBOC clinical personnel and resources due to the lengthy and formal process involved in establishing the clinics, scheduling logistics, and availability of space. Other challenges included the logistics of scheduling in different time zones, and the requirement for dual appointments with the CBOC and the medical center. Retrospective chart review was based on a limited mean follow-up time of 15 months, and assessment of long-term CVT effects on LTSS and caregiver distress and other health care outcomes and utilization were not able to be assessed.

## 5. Conclusions

CVT for dementia consults was well received and may be helpful in supporting dementia caregivers to navigate the healthcare system and assisting to provide LTSS for high-need older adults in rural areas.

## Figures and Tables

**Table 1:** CVT Encounters (March 2015–March 2018).

CBOC location and number of encounters (N = 45)	
Tullahoma	7
Roane County	8
Chattanooga	6
Cookeville	11
McMinnville	13
Mean distance from consult clinic 103 (76–148) miles	
Satisfaction with CVT visit 44 (98%)	
Transportation miles saved 8770	
Diagnoses	
No Dementia 9 (20%) mean age 71 (54–85) years	
Comorbidities (3 PTSD, 2 Depression, 2 Bipolar) 78%, 1 each CFH, DM	
Dementia 36 (80%) mean age 77 (63–100) years	
Medical co-morbidities (11 DM, 8 CHF, 8 Depression, 5 CVA) 89%	
Dementia Patients N = 36	
Dementia type: Alzheimer’s 31 (86%), Vascular predominant 5 (14%)	
Stage: Early 16 (17%), Mid 18 (50%), Late 12 (33%)	
Mean # meds 8 (2–20)	
Two or more ADL deficits 16 (44%)	
Recommendations: LTSS 23 (64%), medications 13 (36%) diagnostic testing 8 (22%)	
Follow-up interval 15 (1–36) months	
Transitioned to higher level of care 7 (19%)	
Advance care planning in place 16 (44%)	
Died 7 (19%) mean 10.2 months following consultation	
Caregivers: spouse 70%, other family members 30%	
Advanced caregiver distress present 17 (47%)	

Legend: ADL activities of daily living; CBOC community-based outpatient center; CHF congestive heart failure; DM diabetes mellitus; CVA stroke; CVT Clinical Video Telehealth LTSS home and community-based long-term care support services: PTSD post-traumatic stress disorder.
